# Jewish Physicians in Hamburgh

**Published:** 1845-05

**Authors:** 


*Jewish Physicians in Hamburgh.*—In consideration of the active and generous conduct of the Jews of Hamburgh, and especially of the great banker, Solomon Heine, on occasion of the fire in that city in 1842, their condition has been ameliorated. Before, the laws weighed heavily upon them, as every where else in Germany. For a long while, the Jews of Hamburgh have been rigidly restricted to commerce and to the exercise of the *medical profession.* Now, the Council of Ancients propose to open to them all trades and professions.

What a low estimate, it would appear, has been made of medicine, by the enlightened people of Hamburgh! If the oppressed Israelites studied any science, it must be medicine—as much as to say, it is only a mean occupation, and very well accords with their character and political circumstances. Associated with degradation as medicine is, when practised by a Jew, some of the brightest luminaries of the last century were of that despised, persecuted race. Those who have read Dr. Dunbar's translations of the early efforts and claims of the Jewish physicians of Europe, know very well our indebtedness to them, notwithstanding the low estimate in which they are held by the authorities of Hamburgh.

*Boston Med. and Surg. Jour.*

